# Which Visual Modality Is Important When Judging the Naturalness of the Agent (Artificial Versus Human Intelligence) Providing Recommendations in the Symbolic Consumption Context?

**DOI:** 10.3390/s20175016

**Published:** 2020-09-03

**Authors:** Kyungmi Chung, Jin Young Park, Kiwan Park, Yaeri Kim

**Affiliations:** 1 Department of Psychiatry, Yonsei University College of Medicine, Yongin Severance Hospital, Yonsei University Health System, Yongin 16995, Korea; chungkyungmi@yuhs.ac (K.C.); empathy@yuhs.ac (J.Y.P.); 2 Institute of Behavioral Science in Medicine, Yonsei University College of Medicine, Yonsei University Health System, Seoul 03722, Korea; 3 SNU Business School, Seoul National University, Seoul 08826, Korea; kiwanp@snu.ac.kr; 4 Department of Marketing, Business School, Sejong University, Seoul 05006, Korea; 5 Department of Digital Marketing, School of Management, Sejong Cyber University, Seoul 05000, Korea

**Keywords:** artificial intelligence, symbolic consumption, human uniqueness area, identity of recommendation agent, neuromarketing, electroencephalogram (EEG), event-related-potential (ERP), visual modality, perceived naturalness

## Abstract

This study aimed to explore how the type and visual modality of a recommendation agent’s identity affect male university students’ (1) self-reported responses to agent-recommended symbolic brand in evaluating the naturalness of virtual agents, human, or artificial intelligence (AI) and (2) early event-related potential (ERP) responses between text- and face-specific scalp locations. Twenty-seven participants (*M* = 25.26, *SD* = 5.35) whose consumption was more motivated by symbolic needs (vs. functional) were instructed to perform a visual task to evaluate the naturalness of the target stimuli. As hypothesized, the subjective evaluation showed that they had lower attitudes and perceived higher unnaturalness when the symbolic brand was recommended by AI (vs. human). Based on this self-report, two epochs were segmented for the ERP analysis: human-natural and AI-unnatural. As revealed by P100 amplitude modulation on visual modality of two agents, their evaluation relied more on face image rather than text. Furthermore, this tendency was consistently observed in that of N170 amplitude when the agent identity was defined as human. However, when the agent identity was defined as AI, reversed N170 modulation was observed, indicating that participants referred more to textual information than graphical information to assess the naturalness of the agent.

## 1. Introduction

Artificial intelligence (AI) recommendation systems are all around us [1]. In both offline and online shops, they reduce consumers’ effort in searching for products and services. In some cases, such as when a person is searching for utilitarian solutions or answers, AI recommendations may be welcomed (e.g., “What material can withstand the most heat?”). However, would a consumer feel the same way if AI evaluated their needs in a uniquely human area? Previous studies have found that recognizing one’s ego represents a uniquely human capacity [2] because understanding one’s ego serves as the first step to autonomy, and to free will. Thus, when an algorithm is perceived to have autonomy, people may feel that this represents a threat to humanity [3]. Therefore, we first aim to determine what constitutes a uniquely human area in consumer behavior. We find a clue to the answer in the realm of symbolic consumption—expressing one’s personality through products or services [4]. The second concern relates to the core research question of this study: How will consumers react when AI invades the realm of uniquely human consumption behavior? To answer this question, we conducted a study through a neurologically grounded approach by collecting electroencephalogram (EEG) data and subjective rating evaluations from the participants.

### 1.1. Symbolic Consumption and Human Uniqueness

Consumer purchase motivations are classified into three subcategories: functional, experiential, and symbolic needs [5]. According to previous studies, each motivation employs a different type of information processing. When consumers are motivated by functional needs, utilitarian benefits are considered a key factor in making consumption decisions. In contrast, experiential motivation is related to hedonic benefits, wherein the affective process is activated [6]. Symbolic consumption motivation differs from the first two types in that it originates from social cognition [5,7]. People seek out products and services that can enhance their self-image and social status when stimulated with symbolic motivation. The core motivation of symbolic consumption is the desire to express one’s own characteristics [8]. Thus, understanding one’s ego and admirable target-self serves as a prerequisite to understanding symbolic consumption [9,10,11]. With this perspective, we find that recognizing one’s ego is a uniquely human area, indicating that a non-human entity—even one that features AI—cannot learn the essence of ego even through deep or machine learning [2]. For instance, AI would not ask the question, “What is ego?” and feel concerned about how to improve its social or personal self within social relationships.

### 1.2. AI Recommendations in Symbolic Consumption

AI is defined as “a non-human entity that is autonomous, interacts with its environment, and adapts itself as a function of its internal state and its interaction with the environment” [12]. Nowadays, it is typical to encounter AI features in a store that provide recommendations for consumers’ choices. However, some people still prefer to obtain recommendations only from a human salesperson, especially in some specific areas. We contend that the symbolic consumption context may be an area where people particularly do not want to feel imposed upon by a non-human entity because symbolic motives represent the inherent realm of humans. This contention is consistent with the “threat to distinctiveness” hypothesis [13]. Studies approach human need for distinctiveness among other groups, with a focus on anthropomorphic features of robots [14]. For example, an android robot with a humanlike appearance poses a greater threat, compared to a robot with mechanical appearance. However, the current study does not simply undertake an investigation into a superficial appearance-based threat, but rather a more fundamental distinctive motive by incorporating the capabilities of uniquely human areas [15,16], are closely linked to the human ego and consumption fueled by symbolic motivation. 

### 1.3. Background on AI Recommendation Agents

The previous literature on the perspectives of consumer attitudes toward AI has been selectively developed. Depending on the information provided, consumers’ attitude toward an AI agent may be either positive or negative. Specifically, people cease their reliance on an algorithm when they notice a mistake in it [17]. Conversely, they increase their reliance on the algorithm when the information provided in the output of the algorithm is modifiable [18]. When people are informed that the AI possesses learning capabilities, they show increased intent of adopting the AI recommendations even in a situation in which the reason for which the action is conducted is emphasized [19]. However, so far, few studies have focused on the decision-processing phase or how consumers’ attitudes or intentions are established. Thus, the current study focuses on which factors affect consumers’ decision-making process regarding an AI recommendation agent. By providing both visual and textual information on recommendation agents and varying their identity as human versus AI, we investigate the importance of visual modality during the decision-making process.

### 1.4. Approach to Measuring Consumer Behavior in Agent-Recommended Symbolic Consumption

Apart from the importance of visual information on a recommendation agent that is associated with the type and modality of agent’s identity in a symbolic consumption context it is difficult to measure individual differences in both explicit and implicit attitudes or perceptions toward intelligent, agent-based systems for personalized recommendations in commerce in a quick, accurate, and cost-efficient manner. To deal with the challenge of understanding consumer behavior and the decision-making process, more attention has been paid to applying EEG measurement and the event-related potential (ERP) analysis method in marketing research [20]. According to the findings of a number of event-related potential (ERP) studies [21,22,23,24,25,26], the notion that processing faces and characters involves categorically different neural mechanisms has been well supported, particularly as indicated by the amplitude and lateralization of P1 and N170 components. From the domain-specific view of face processing, the earliest categorical difference is observed in the P1 component, followed by the N170 component, and the amplitude of the N170 is greater for faces relative to characters, while the hemispheric distribution of the N170 is bilateral for faces and more left-lateralized for characters. Furthermore, previous studies have revealed that the left-lateralized N170 component for characters is consistently reported in different languages, such as Chinese [26,27], English [21,22,28], Japanese [25], Korean [23], and Roman characters [27]. Given that the amplitude and lateralization of these ERP components produce replicable outcomes to reveal the categorical difference between the two types of agents’ identity modalities, it is expected that the neurophysiological markers outlined above will provide an intriguing opportunity for measuring the discrepancy between (male) consumers’ explicit and implicit attitudes toward two recommendation agents and predicting their symbolic brand choice across agents.

### 1.5. Objectives

This study aimed to examine how the type and modality of a recommendation agent’s identity, as independent variables, would affect male university students’ (1) early ERP responses between the text- and face-specific scalp locations and (2) self-reported responses to agent-recommended symbolic brand, the dependent variable, in terms of evaluating the naturalness of the virtual agents in a symbolic consumption context. From the theoretical perspectives of previous studies, we assume that human agent-recommendations will be perceived as more natural than AI agent recommendations, particularly within the context of symbolic consumption in relation to one’s own self-image and social status. Based on this fundamental assumption of the current study, the research question is as follows: What is the important visual modality when assessing the naturalness of the agent (AI vs. human) providing recommendations in a symbolic consumption context?

## 2. Materials and Methods

### 2.1. Experimental Design

To answer the research question, the experiment had a 2 (identity type: human vs. AI) × 2 (identity modality: text vs. face image) within-subjects factorial design with the EEG and subjective measurement. As an implicit measure, the text- and face-specific ERP responses, P1 and N170 amplitudes, were measured at the two regions of interest, particularly the lateral posterior P7 and P8 electrode sites, respectively. As an explicit measure, the subjective evaluation investigated the attitude toward a symbolic brand recommended by two different agents and the perceived naturalness of the two agents’ recommendations in symbolic consumption. In this study, we first implemented a manipulation check to choose the appropriate visual stimuli, followed by a main EEG experiment.

### 2.2. Participant Recruitment

For the main EEG experiment, a total of 30 men were recruited from a list of university students who signed up for an online community site named everytime (www.everytime.kr), where our recruitment advertisement was posted to provide them with an online link to apply for participation in this experiment along with brief information on the study protocol. All participants were undergraduate or graduate students at universities in Seoul, South Korea, and were right-handed male students. We selected male participants to avoid biases from sex specificity of symbolic consumption [29]. Further, in accordance with the inclusion and exclusion criteria for enrollment, (1) those whose motivational drive for consumption depended more on the functional values of products or services than on their symbolic values or (2) those who did not understand the concept of symbolic consumption were excluded from the study. To determine whether an individual’s consumption is more motivated by symbolic needs than by functional ones, all participants were asked to complete a questionnaire on features they considered important for a brand [30,31].

Of 30 participants who provided written informed consent, 28 were eligible to participate in the experiment because the other two reported that they made consumption decisions based on functional or product-related needs. After the completion of the entire experimental procedure and debriefing, they were paid 10,000 KRW for their participation. This study was approved by the institutional review board (IRB) of Seoul National University in Seoul, South Korea (IRB No. 1912/003-001).

### 2.3. Visual Stimuli and Manipulation Check

**Figure 1:**
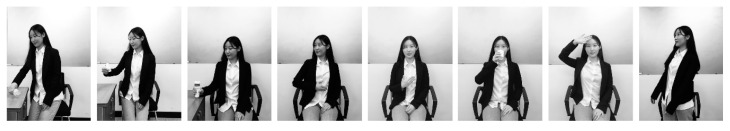
All eight agent images used in manipulation check for determining whether there were significant differences between mean scores for ratings of their attractiveness and naturalness across agent identity.

As visual stimuli displayed in an ERP paradigm, eight still images of a Korean woman (Figure 1), who performed eight different actions [32,33] and engaged in head tilts mimicking a human-like robot [34], were prepared and repeatedly used to represent human and human-like AI agents, both of which were only distinguished by word labels placed on the top of the last screen: human and AI. Furthermore, a manipulation check was administered to rule out the possibility that participants would evaluate the naturalness of the two recommended agents based only on their actions and appearance, not based on the symbolic consumption context. In the manipulation check, 31 male participants who are all right-handed (age range, 19–39 years; *M* = 26.52, *SD* = 4.34) were recruited from Sejong University in South Korea. After a set of eight images was randomly presented twice in a counterbalanced order (agent, human vs. AI), attractiveness (1, not attractive at all; 7, very attractive) and naturalness (1, not natural at all; 7, very natural) were rated on 7-point scales repeatedly with appropriate anchors within the same respondents, assuming that either of the two different identities was assigned to each image in the set. According to the result of a paired samples t-test, there was no significant difference in the mean scores for the subjective ratings of the two agents: (1) attractiveness (*M_human_* = 4.94, *SD_human_* = 1.00 vs. *M_AI_* = 4.71, *SD_AI_* = 1.07; *t*_30_ = –1.27, *p* = 0.21) and (2) naturalness (*M_human_* = 4.16, *SD_human_* = 1.55 vs. *M_AI_* = 4.42, *SD_AI_* = 1.57; *t*_30_ = 1.25, *p* = 0.22). Of the eight images, three, which were perceived as the most natural and attractive in rank by the 31 men, were selected for use in the main EEG experiment.

### 2.4. Equipment

In the main EEG experiment, we used an E-Prime version 2.0 software (Psychology Software Tools Inc.; PST, Pittsburgh, PA, USA) to create our experimental paradigm to deliver visual information on a recommendation agent associated with the agent’s identity type (human vs. AI) and modality (text vs. image) in a symbolic consumption context. We used PST’s E-Prime Extensions for Net Station (EENS) version 2.0 software to record psychological and behavioral responses to visual stimuli in the given task. The experimental paradigm was displayed on a Dell Latitude E6430 laptop computer (Dell, Round Rock, TX, USA) with a 14-inch screen resolution of 1366 × 768, and a refresh rate of 60 Hz. EEG data were acquired via a GES 400 system (Electrical Geodesics Inc.; EGI-Philips, Eugene, OR, USA) using the EGI-Philips’ Net Amps 400 amplifier, 128-channel HydroCel Geodesic Sensor Net (HCGSNs), and Net Station version 5.4.2 software (i.e., Net Station Acquisition, Review, and Tools) run by an Apple MacBook Pro (Apple Inc., Cupertino, CA, USA).

### 2.5. Experimental Paradigm and Task Procedure

**Figure 2:**
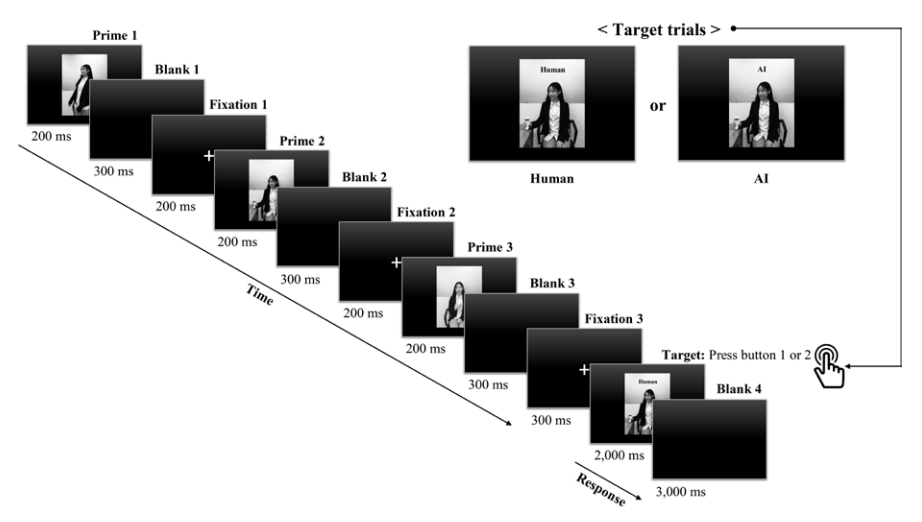
Event-related potential (ERP) experimental paradigm presented during the electroencephalogram (EEG) recording.

After the three images of visual stimuli were selected in the manipulation check, the experimental paradigm was composed of a practice block of four trials (two human and two AI trials) and a main block of 60 trials (30 human and 30 AI trials). The two types of target trials presented—human and AI trials—were randomly distributed. In both trials, a prime procedure with three agent images as primes (200 ms), which were followed by a blank (300 ms) and a fixation cross (200 ms for the presentation of the first and second images and 300 ms for that of the third image), and one of the three agent images and one of the two words (human or AI) as targets (onset of target trial: 2000 ms), followed by a blank (3000 ms), was employed (Figure 2). Furthermore, the same agent images with different agent identity information in text (human vs. AI) in the upper center of the screen were presented for the two target trials. In each of the 60 target trials lasting for 5000 ms, participants were asked to indicate whether they would consider it natural or unnatural for the displayed agent to provide them with recommendations in a symbolic consumption context, making this selection by pressing the keyboard button 1 or 2, respectively, with their right hand as quickly and accurately as possible. The behavioral responses were recorded only when participants responded within 5000 ms after the onset of the target stimuli. Participants received no feedback on their reaction times or whether their responses matched expectations, but those behavioral responses were recorded via the E-Prime software for preprocessing EEG signals.

Before starting the ERP paradigm, participants were asked to not only perform a visual task where they first had to imagine a symbolic product to purchase on the recommendation of virtual agents to be presented on the screen but also continue to respond to each trial as prompted.

### 2.6. Subjective Measures

#### 2.6.1. Consumption Type for Brand

To ensure that this study sample was more motivated by symbolic needs than by functional or product-related ones in terms of participants’ consumption decisions, we adapted six-item scales developed to assess a brand’s functional or symbolic association with consumers [30,31]. Before starting the EEG recording, the 28 participants were required to indicate which feature they considered most important in a brand: (1) functional (quality of the product, economic efficiency of the product, and function of the product) or (2) symbolic (representing their social status, identity, and personality). Each item was scored on a 7-point scale anchored by 1 (not important at all) and 7 (very important), and the mean scores were calculated across two features to be regarded as important consumption motivation.

#### 2.6.2. Attitude Toward Symbolic Brand Recommended by Different Agents

To compare the difference in attitude toward the symbolic brand suggested by the two recommendation agents, we used three seven-point semantic differential (SD) scales: (1) positive–negative, (2) good–bad, and (3) favorable–unfavorable. After completing the EEG recording, all participants were asked to indicate their attitude toward the symbolic brand when recommended by a human or AI agent [35]. Each scale was scored from 1 to 7, and attitudes were represented by each participant’s mean score on the three SD scales for the two different recommendation agents.

#### 2.6.3. Perceived Naturalness of Different Agents’ Recommendations in Symbolic Consumption

To evaluate the perceived naturalness of recommendations provided by the two different agents in a symbolic consumption context, six-item scales were adapted from a study by Gray and Wegner [15]. Participants were prompted to indicate their responses to the following statements: (1) I felt uncomfortable about the (human or AI) recommendation in symbolic consumption; (2) I felt unpleasantly about the (human or AI) recommendation in symbolic consumption; or (3) I had an awkward feeling about the (human or AI) recommendation in symbolic consumption. All items were rated on a 7-point scale from 1 (not very likely) to 7 (very likely), and each of the three items was separately averaged for a human or AI agent.

### 2.7. Objective Measures

#### ERP Components: P100 and N170

P100 is a prominent ERP component involved in early sensory processing, particularly the visual attention processing of the parietal-occipital regions, and equally responsive to all categories. The P100 is a positive-going waveform in peak amplitude that occurs approximately 100 ms after post-stimulus onset. N170 is also an early visual ERP component involved in the category-specific encoding processing of characters and faces. Text- and face-specific N170 components are characterized by a negative-going waveform in peak amplitude that occurs approximately 170 ms after the onset of stimulus and observed at the left and right parietal-occipital regions.

In this study, the amplitude of both early ERP components in response to visually presented stimuli, particularly within in the time window of P1 (70–130 ms) and N170 (150–200 ms), was measured at the P7 and P8 electrode sites. Although the P100 reflects a stimulus category effect, which is associated with low-level stimulus characteristics [36], top-down attention processes can affect P100 amplitude [37], indicating that the category-specific ERP responses can be observed. The text-specific visual processing was identified at the P7 electrode, and the face-specific visual processing was identified at the P8 electrode. To analyze the ERP responses based on the hypothesis that human agent-recommendations would be perceived as more natural than AI agent recommendations in the context of symbolic consumption, the ERP responses in which participants perceived human agent as natural and AI agent as unnatural were detected throughout the phase of segmentation during the EEG data preprocessing and used for the ERP analysis.

### 2.8. EEG Data Acquisition and Preprocessing

For recording EEG signals, an electrolyte solution of potassium chloride (11 g), baby shampoo (5 mL), and water (1 L) was made using the provided spoons for each substance; then, the 128-channel HCGSN with sponge inserts was soaked in the electrolyte solution for 10 min. While waiting, the experimenter measured the circumference of each participant’s head to determine the appropriate size of the HCGSN, and the point to which the Cz electrode would be placed as a reference was marked on their scalp using a colored pencil to aid in the correct placement of the HCGSN. Following the HCGSN application, adjustments were made to each electrode until the impedance of all electrodes remained below 50 kΩ to ensure an optimal signal-to-noise ratio for the high-input impedance amplifier [38]. All EEG signals were referenced to a single vertex electrode Cz, recorded with a 0.01 to 400 Hz analog band-pass filter, and digitized at 1000 samples per second online.

During preprocessing, the data were filtered offline with a 0.3 to 30 Hz digital band-pass filter and segmented into epochs ranging from 200 ms before to 1000 ms after the onset of each of the two stimulus conditions: (1) human-natural (the response was 1 if the agent’s identity was human in a symbolic consumption context) and (2) AI-unnatural (the response was 2 if the agent’s identity was AI in the same context). Based on a timing evaluation to guarantee the accuracy of visual stimuli presentation in E-Prime and EENS, each segment was offset by the average offset value of 11 ms. Based on the programmed algorithm of EGI-Philips’ Net Station Tools, artifacts such as eye blinks, eye movements, and bad channels were identified as follows. If a channel was bad for more than 20% of the segments, the channel was marked as bad for all segments. Segments were marked as bad if they contained (1) more than 10 bad channels (maximum–minimum >200 μV for the entire segment, with a moving average of 80 ms); (2) eye blinks (maximum–minimum >140 μV, with a moving average of 80 ms); or (3) eye movements (maximum–minimum >55 μV, with a moving average of 80 ms). Then, bad channel replacement was performed. For the individual ERP analysis, the data were averaged for individual participants and baseline-corrected for the 200 ms pre-stimulus period; then, all channels were re-referenced offline to the average reference. For the whole ERP analysis, the baseline-corrected data were utilized for grand averaging, baseline-corrected for the same pre-stimulus period, and ultimately re-referenced to the average reference using the data from all 28 participants.

To identify the presence of the P1 and N170 components, we determined the electrodes showing the largest amplitudes for each of the ERP deflections of interest using the grand average waveforms. For the statistical analysis, the adaptive mean values of the P1 and N170 amplitudes at the P7 (left) and P8 (right) electrode sites were extracted from the separate and grand average waveforms within the following time windows: P1 (70–130 ms) and N170 (150–200 ms).

### 2.9. Statistical Analysis

In this study, all statistical analyses were performed using PASW Statistics 18.0 software (SPSS Inc. Chicago, IL, USA). As the sample size (*N* = 27) was smaller than 2000, a Shapiro–Wilk test was used to evaluate the normal distribution of the data; thus, employing an appropriate statistical analysis method. While the normality assumption of the ERP data was met (*p* < 0.05), that of the self-reported data was violated. For the within-participant comparison of the self-reports on the recommendations provided by two different virtual agents, the Wilcoxon signed-rank test was conducted as a non-parametric statistical test. To reveal the ERP evidence for the effect of the recommendation agent’s identity type and identity modality on the perceived naturalness of the two different virtual agents, a two-way repeated measures analysis of variance (RM-ANOVA) was conducted as a parametric statistical test. When an interaction effect was found significant, a planned post hoc test was conducted using the Bonferroni correction method. To determine how much of an effect independent variables had on the dependent variables, we used the following cutoff values to interpret our results: (1) large: 0.14 or more; (2) medium: 0.06 or more; and (3) small: 0.01 or more.

## 3. Results

### 3.1. Participant Characteristics

Following the exclusion criteria for data analysis, one participant, whose data file was not accessible via both Net Station Tools (Electrical Geodesics Inc; EGI-Philips, Eugene, OR, USA) and MATLAB software (R2016b; MathWorks, Inc, Natick, Massachusetts, USA), was excluded, and therefore a total of 27 male university students aged 19 to 40 years (*M* = 25.26, *SD* = 5.35) were finally enrolled in this study. All enrollees were right-handed, with normal or corrected-to-normal vision. According to the self-report on features considered important in a brand, we determined that individuals’ consumption had been more motivated by symbolic needs (*M* = 5.47, *SD* = 1.01) than by functional needs (*M* = 4.62, *SD* = 1.54) in this study sample (*t*_26_ = – 1.96, *p* = 0.06).

### 3.2. Subjective Evaluation of the Difference in Recommendation Agents Supporting Symbolic Consumption

#### 3.2.1. Attitude Toward Symbolic Brand Recommended by Different Agents

The Wilcoxon signed-rank test indicated that participants rated the AI agent-recommended symbolic brand (mean rank = 14.48; *M* = 3.83, *SD* = 0.84) more negatively than the human agent-recommended symbolic brand (mean rank = 10.25; *M* = 3.05, *SD* = 0.68, *Z* = –2.90, *p* = 0.004).

#### 3.2.2. Perceived Naturalness of Different Agents’ Recommendations in Symbolic Consumption

According to the results of the Wilcoxon signed-rank test, the AI agent’s recommendation (mean rank = 12.97; *M* = 3.57, *SD* = 0.88) was rated as more unnatural than that of the human agent (mean rank = 13.07; *M* = 2.91, *SD* = 1.27) in symbolic consumption advice (*Z* = –1.91, *p* = 0.056).

### 3.3. ERP Evidence on Associations with Visual Information on Recommendation Agent’s Identity Type and Modality in Symbolic Consumption and Perceived Naturalness

**Figure 3:**
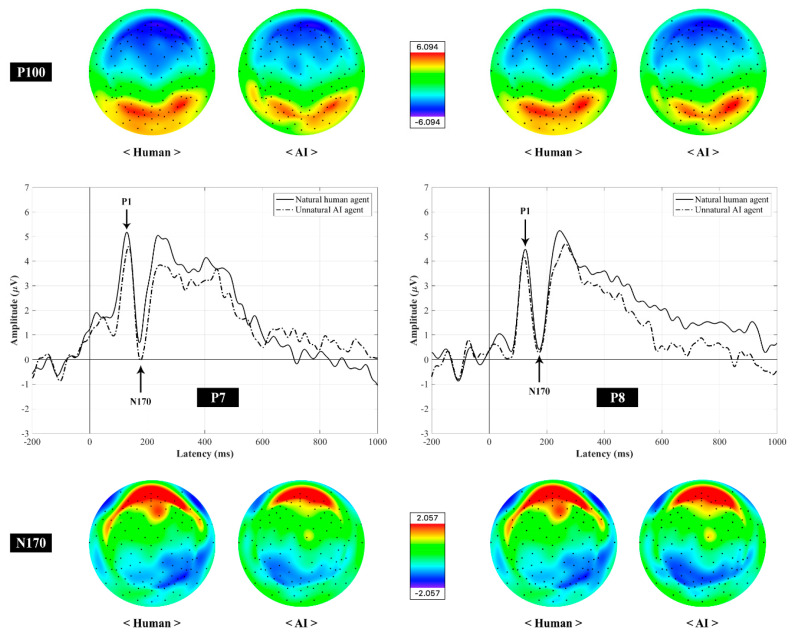
Grand averaged ERP waveforms and topographical maps of P1 (P100) and N 170 recorded at P7 and P8 electrode sites, elicited by two different agent stimuli associated with images and words.

Figure 3 shows that both human and AI agents’ images and words elicited clear P1 and N170 components in topographical distribution on the scalp and their grand-averaged ERP waveforms.

#### 3.3.1. P1 Component

**Figure 4:**
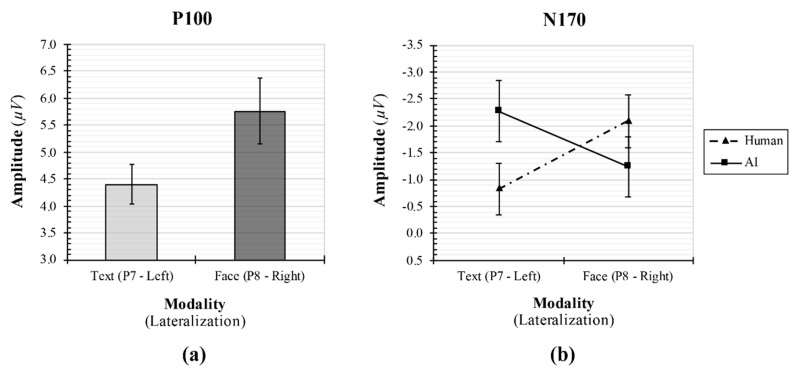
(**a**) Main effect of agent’s identity modality on changes in P1 (P100) amplitude and (**b**) interaction effect of agent’s identity type and modality on changes in N170 amplitude.

The result of the two-way RM-ANOVA showed a significant main effect of agent’s identity modality on change in P1 amplitude (*F*_1, 26_ = 5.20, *p* = 0.03, *η_p_*^2^ = 0.17), such that the P1 amplitude at the right posterior electrode P8 (*M* = 5.76, *SE* = 0.62) was larger than that at the left posterior electrode P7 (*M* = 4.41, *SE* = 0.36; Figure 4a). Hence, the modality effect was greater for the face image modality than for the text modality. However, no main effect of agent’s identity type (*F*_1, 26_ = 0.43, *p* = 0.52, *η_p_*^2^ = 0.02) and no interaction between the effect of two independent variables (*F*_1, 26_ = 1.66, *p* = 0.21, *η_p_*^2^ = 0.06) on changes in P1 amplitude emerged.

#### 3.3.2. N170 Component

The two-way RM-ANOVA yielded a statistically significant interaction between the effect of agent’s identity type and modality on change in N170 amplitude (*F*_1, 26_ = 6.34, *p* = 0.02, *η_p_*^2^ = 0.20), indicating that when the agent’s identity was human, the face-specific N170 amplitude (*M* = –2.09, *SE* = 0.49) exhibited an increased tendency, compared to the text-specific N170 amplitude (*M* = –0.83, *SE* = 0.48) (*p* = 0.057 with a Bonferroni-corrected critical *p*-value of 0.0125). The text-specific N170 amplitude showed a tendency toward a greater increase in the AI agent (*M* = –2.27, *SE* = 0.57) than in the human agent (*M* = –83, *SE* = 0.48) (*p* = 0.035 with the Bonferroni-corrected *p*-value of 0.0125; Figure 4b). However, there were no significant main effects of agent’s identity type (*F*_1, 26_ = 0.32, *p* = 0.58, *η_p_*^2^ = 0.01) and modality (*F*_1, 26_ = 0.06, *p* = 0.82, *η_p_*^2^ = 0.002) on changes in N170 amplitude.

## 4. Discussion

This study aimed to reveal (1) the ERP evidence for the effect of the recommendation agent’s identity type and modality on the perceived naturalness of the two different virtual agents and (2) the subjective ratings of agent-recommended symbolic brand among male university students. The current study was based on the hypothesis that human agent-recommendations would be perceived as more natural than AI agent recommendations in the context of symbolic consumption in relation to one’s own self-image and social status. Following this, we attempted to test this hypothesis via explicit and implicit measures and answer our own research question by segmenting participants’ behavioral responses into two epochs, such as human-natural and AI-unnatural, and then conducting a series of the ERP analysis. Based on the following findings of this study, we determined which visual modality would be considered as important when assessing the naturalness of the agent (AI vs. human) providing symbolic consumption-related recommendations.

The main findings of the current study are as follows. As hypothesized, the self-reported results showed that they had significantly lower attitudes and perceived higher unnaturalness when the symbolic brand was recommended by an AI agent compared with a human agent. As revealed by a main effect of agent’s identity modality on change in P1 component as a human visual evoked potential, the P1 amplitude at the right posterior parietal cortex (P8) was greater than that at the left posterior parietal region (P7), indicating that participants depended more on graphical information, such as face, motion, and appearance, than on textual information written as human or AI when judging the naturalness of the recommendation agent. As the earliest ERP response to visual stimuli, the values of P1 amplitude reveal that the graphical information led participants to draw their attention than the textual information did, regardless of agent’s identity type. Furthermore, an interaction between the effect of agent’s identity type and modality and change in N170 component, which was considered as face-specific at the right posterior parietal site and text-specific at the left posterior parietal region [21,22,23,25,26], was observed. Particularly when the agent identity was defined as human, this tendency in the P1 component was also consistently observed in the N170 component. However, when defined as AI, reversed N170 modulation was observed, indicating that participants referred more to textual information than graphical information to assess the naturalness of the agent. Consistent with numerous previous studies employing different languages [21,22,23,25,26], the notion that the category-specific processing of faces and characters was based on distinct neural mechanisms was evidenced by the P1 component, followed by the N170 component in this study. In this regard, both ERP components, which provide the evidence for the earliest categorical difference in processing of graphical and textual information on recommendation agent, could serve as a promising neurophysiological marker in predicting the effect of visual modality on consumer decision.

### 4.1. Theoretical Implications

The current study makes some critical theoretical and managerial contributions. First, despite the substantial attention that AI has garnered, the literature that has explored consumers’ perceptions of AI has relied mainly on self-reported rating methods of research [39]. However, the current study utilized EEG data, rendering it the first attempt to make a methodological contribution to consumers’ perception of AI.

Second, most of the research pertaining to AI appearances is theoretically based on the uncanny valley [40], which postulates that when a robot’s appearance becomes increasingly similar to a human’s, it eventually reaches an uncanny point that people find unsettling. The current study differs from previous studies in that the appearance of the AI and human was presented as similar and the visual appearance was controlled in the experiment. Thus, by suggesting the possibility that uncanniness does not stem from visual appearance, we make a significant contribution to the literature. Thus, we assert that the uncanny feelings people may experience toward robots are not always due to visual appearances but can also be triggered by situational or textual information [15].

### 4.2. Managerial Implications

The results of the current study help practitioners in the marketing field. Taking into account the fact that P1’s tendency of human recommendation agents’ suggestions to participants continued into N170, the participants processed the suggestions of human agents as being natural. When the recommendation agent was framed as AI, they relied more on textual information. Thus, it is one potential avenue to investigate the possibility that the information conveyed through text will determine whether AI suggestions on symbolic consumption are considered natural.

Instead of conveying through text that the recommendation agent is AI, conveying the agent’s identity as a human character could therefore serve as one way of reducing consumers’ repulsion. In addition to grafting a human identity onto the AI agent, conveying to consumers that the algorithm is capable of improvement through machine learning [19] or displaying signs that the AI agent has a lower probability of making errors than humans, may allow the agent to come across as more approachable to consumers, instead of the current marketing communication method of characterizing the agent as AI through text [41]. Thus, we believe this study can serve as a good starting point from which these concepts can be improved upon for future research.

Furthermore, the majority of ERP experiments are extremely long; thus, as an experiment unfolds, the participants familiarize the experimental protocols, and the observations obtained after this adaptation are all recorded as data. However, in the case of advertisements, people’s short-term perceptions after exposure are more important. In this study, the experimental protocol reflects these considerations, and the EEG data collection period was made as short as possible. This study makes significant efforts to ensure the experimental methods are as applicable to the real world as possible, in contrast to most laboratory experiments, which are conducted with the idea that real-world applications differ from the experimental results. Thus, our method of analyzing people’s EEG data is analogous to what people process information after exposure to advertisements in real life.

### 4.3. Limitations and Further Research

This study provides critical theoretical and practical implications, but there are a few experimental limitations. First, the sample consisted of only Korean individuals; thus, AI perceptions cannot be generalized across people from other countries based on this study. Every country’s technological advancements progress at a different rate; thus, we postulate that the technological perceptions of people in other countries could be extremely different [42]. Additionally, the exclusion of women from the sample could be both a limitation and strength as, in the case of symbolic consumption, women’s perceptions are generally better understood [43]. Thus, previous neuro-studies were conducted based on female participants [44,45]. However, the gender effect in symbolic consumption has been proved in that female consumers have higher identification in symbolic brands and consider greater aspects of value, including hedonic, unique, and status than male consumers [46]. Thus, the need for neuro-research in understanding male participants in symbolic consumption domain has been increased. As a result, the current study was conducted with male participants, which lowers the barrier for future research, alleviating the limitations of the current literature on symbolic consumption.

Second, the current experiment only involved symbolic consumption situations. For more comprehensive results, conducting a comparative study with functional consumption motivation situations would yield more accurate data. However, we screened for participants with greater symbolic consumption motivation during our recruitment process, and thus this limitation was somewhat alleviated. Although the difference between two motivations among our screening questions yielded a marginally significant result (*p* = 0.062) and could be seen as a limitation, conducting this experiment with a larger sample size in the future would alleviate this limitation. Furthermore, functional and symbolic motivations are intertwined in the purchase of any product, and the comparative advantage is filtered out; thus, this limitation is fairly well resolved. Nevertheless, the limitation of not having directly compared functional vs. symbolic consumption should be addressed through future research.

Finally, the possibility that participants have differing mental perceptions when they process the concept of “AI,” as opposed to the more common “robot,” must be considered. Different results could emerge if the agents were framed as robot or humanoid android, which must be explored in a follow-up study.

## 5. Conclusions

The importance of visual modality (image vs. text) when assessing the naturalness of the agent providing recommendations in a symbolic consumption context differs depending on the agent’s identity. When the agent’s identity is described as human, the information provided by the picture plays a more critical role in individuals’ reaction to the agent. However, when the agent is characterized as AI, the dependence on textual information becomes stronger. Although this finding cannot fully explain whether people resist AI involvement in the symbolic consumption process, it appears clear that the factors affecting consumers’ decisions vary depending on how marketers describe the AI technology they utilize.
